# Badapple: promiscuity patterns from noisy evidence

**DOI:** 10.1186/s13321-016-0137-3

**Published:** 2016-05-28

**Authors:** Jeremy J. Yang, Oleg Ursu, Christopher A. Lipinski, Larry A. Sklar, Tudor I. Oprea, Cristian G. Bologa

**Affiliations:** Translational Informatics Division, Department of Internal Medicine, University of New Mexico School of Medicine, Albuquerque, NM 87131 USA; 10 Connshire Drive, Waterford, CT 06385-4122 USA; Department of Pathology, Center for Molecular Discovery, University of New Mexico School of Medicine, Albuquerque, NM 87131 USA

**Keywords:** Drug discovery informatics, High-throughput screening (HTS), Compound promiscuity, Molecular scaffolds, Statistical learning

## Abstract

**Background:**

Bioassay data analysis continues to be an essential, routine, yet challenging task in modern drug discovery and chemical biology research. The challenge is to infer reliable knowledge from big and noisy data. Some aspects of this problem are general with solutions informed by existing and emerging data science best practices. Some aspects are domain specific, and rely on expertise in bioassay methodology and chemical biology. Testing compounds for biological activity requires complex and innovative methodology, producing results varying widely in accuracy, precision, and information content. Hit selection criteria involve optimizing such that the overall probability of success in a project is maximized, and resource-wasteful “false trails” are avoided. This “fail-early” approach is embraced both in pharmaceutical and academic drug discovery, since follow-up capacity is resource-limited. Thus, early identification of likely promiscuous compounds has practical value.

**Results:**

Here we describe an algorithm for identifying likely promiscuous compounds via associated scaffolds which combines general and domain-specific features to assist and accelerate drug discovery informatics, called Badapple: bioassay-data associative promiscuity pattern learning engine. Results are described from an analysis using data from MLP assays via the BioAssay Research Database (BARD) http://bard.nih.gov. Specific examples are analyzed in the context of medicinal chemistry, to illustrate associations with mechanisms of promiscuity. Badapple has been developed at UNM, released and deployed for public use two ways: (1) BARD plugin, integrated into the public BARD REST API and BARD web client; and (2) public web app hosted at UNM.

**Conclusions:**

Badapple is a method for rapidly identifying likely promiscuous compounds via associated scaffolds. Badapple generates a score associated with a pragmatic, empirical definition of promiscuity, with the overall goal to identify “false trails” and streamline workflows. Unlike methods reliant on expert curation of chemical substructure patterns, Badapple is fully evidence-driven, automated, self-improving via integration of additional data, and focused on scaffolds. Badapple is robust with respect to noise and errors, and skeptical of scanty evidence.

**Electronic supplementary material:**

The online version of this article (doi:10.1186/s13321-016-0137-3) contains supplementary material, which is available to authorized users.

## Background

### Library design, hit selection and stacking the odds

Efforts to streamline, automate, and rationalize drug discovery in the past two decades have embraced automated high-throughput methods, including HTS and combinatorial chemistry. High-throughput is based on the reasonable premise that testing more molecules will result in discovery of more leads and drugs, all things being equal. However, after the documented failure of high-throughput alone to improve productivity, the consensus seems to be that scaling up throughput is not sufficient, and that “all things being equal” requires expert attention such as for library design. This ongoing reality check has prompted a closer examination of high-throughput methods, and several conceptual and methodological advances, including the Lipinski “Rule of 5” (Ro5) [1], the identification of HTS false positives due to reactivity [2], frequent hitters [3] and promiscuous binders [4, 5], the development of the “lead-like” and “drug-like” concepts [6–9] and its influence on molecular complexity [10], the concept of “ligand efficiency” [11] and the development of fragment-based drug discovery [12, 13].

Given appropriately designed chemical libraries [14], HTS campaigns are usually successful by generating lead compounds and knowledge—structure–activity relationships (SAR)—to facilitate subsequent discovery steps. The probability of success depends both on the quantity and quality of the compounds. From a growing awareness of the importance of compound quality, and apparent failures of “assembly line science”, the Ro5 and lead-like concepts have increasingly influenced library design. Notions of compound quality have progressed and should be informed by a deep understanding of the complex intersection of chemical space and biological target space, which may be termed bioactivity space, and in the context of advancing assay methodologies. Since the usual goal of HTS is discovery of molecules biologically active for a specific target under study, it is logical to regard compound quality as relative: hence, targeted libraries. So, a molecule’s “lead-like” or “drug-like” properties, or coordinates in global chemical space, may be useful conceptually and for coarse garbage-filtering, but the improved goal should be compound fitness for a particular assay or class of assays. In particular, failure to recognize and remove promiscuous and similarly problematic compounds can easily lead to expensive false trails, wasted resources, and flawed conclusions. For example, documented aggregators may not exhibit aggregator behavior under different assay conditions; fluorescent compounds may be unfit for fluorescence-based assays, yet fit for others. Therefore, promiscuity, as well as “lead-like” and “drug-like” properties are assay and target dependent, and should be evaluated within a particular context. All false trails can greatly decrease HTS success rates, since typically only a limited number of hits can be pursued.

The proposed method is intended to complement other methods for detection of bioassay “bad actors”. Baell et al. have introduced “Pan-Assay INterference CompoundS” (PAINS) which combines expert curation of chemical substructure patterns (a.k.a. “structural alerts”) with empirical validation. This extensive work builds upon many efforts [2, 3, 15, 16] to design useful sets of patterns for filtering of unwanted compounds, combined with manual analysis. In the PAINS approach, the combination of expert knowledge of chemical patterns with systematic empirical validation is distinctive and promising. Yet, its reliance on manually-curated chemical patterns is a practical shortcoming, especially for novel chemical patterns and problem-classes. Given our long experience with developing chemical patterns, we regard this activity as necessary and valuable, but a considerable and boundless challenge often involving expert debate and difficulty. In contrast, the method presented herein is fully automatic, fully empirical, and focused on *scaffolds*, a central concept in medicinal chemistry. That is, the promiscuous scaffolds are perceived by the algorithm, and somewhat counter-intuitively, *by virtue of being scaffolds*, are generally as meaningful to chemists, or more so, than substructure patterns. This method requires no code revision to accommodate new data, new assay methods, or new compound classes. The generality of its inferences is limited solely by the breadth and accuracy of the data from which those inferences are derived.

Although library design and hit selection are separate tasks, informatically their effects are merged to determine which compounds are carried forward. Bioassays may be part of a coordinated discovery project, but the data may also be utilized for other quite different discovery projects and scientific goals. Bioassays may yield few hits, or many more than follow up capacity. Hence, filtering and ranking for compound promiscuity and other aspects of suitability in powerful and flexible ways is relevant in a variety of scenarios. The literature does reflect increasing interest in informatics-based approaches to analysis of promiscuity, polypharmacology and non-selectivity assessment [17–19]. Yet, this problem is difficult for reasons closely tied to the difficulties in drug discovery itself, especially the diversity in biological target–ligand interaction mechanisms. We expect that to address this challenge, the community will best be served by a variety of computational methods, well informed by cheminformatics, bioinformatics, screening methodology, discovery workflows and scientific contexts, and enabled by well-structured and annotated data resources.

## MLP, UNMCMD and motivation for Badapple

### Necessity, mother of invention

In 2005, the National Institutes of Health (NIH) launched the Molecular Libraries Initiative (MLP) [20] to seek small molecules that modulate biological pathways in novel ways, as a means to explore chemical biology. This resulted in an unprecedented effort to measure and publish bioactivity data, and the development of a unique compound collection, known as the Molecular Libraries Small Molecule Repository (MLSMR). The NIH MLP involved ten screening centers at academic research sites throughout the USA, which have conducted approximately 2500 assays on over 400,000 unique compounds [21, 22]. Focused on the early stages of lead discovery, with emphasis on target identification, assay development, biomolecular screening, hit-to-probe analysis, this initiative resulted in the discovery of 248 [23] “chemical probes” [24, 25]. Compounds tested by the MLP were largely from the MLSMR [26], developed (by BioFocus DPI, under NIH contract) to support this effort by providing suitable libraries for primary screening. The total number of compounds in MLSMR is currently over 390,000. This library of compounds is designed for HTS fitness based on criteria including calculated physicochemical properties such as solubility, and exclusion of reactive groups [27]. Library design is also based on practical considerations: availability in sufficient quantity and purity, and budget constraints. Additional compounds tested in MLP were obtained in various ways, including purchase and synthesis.

The University of New Mexico Center for Molecular Discovery (UNMCMD) has been an MLP academic screening center since 2005 [28], specializing in high-throughput and multiplexed flow cytometry assays, involving both elucidated molecular targets and phenotypic endpoints. A wide variety of biology has been studied, from cancer cell lines, to bead-based assays, to microbiological pathogens. The UNMCMD Screening Informatics Core has been responsible for bioassay data management and analysis in support of project discovery goals. As such, a key task has been to efficiently analyze bioassay results, identify “true hits”, develop SAR models, and assist in selecting hits for follow up, including confirmation and optimization. The need for efficiency can be painfully clear to those involved in such efforts, given ambitious timelines and the high costs in time, money and labor in following up false trails. From numerous experiences with initially encouraging compounds which turned out to be promiscuous, *and which could have been known as frequent hitters by appropriate analysis of existing assay data*, Badapple was conceived [29].

## Results

### Badapple algorithm

#### Bayes, baseball, and Badapple

The Badapple approach and algorithm detects patterns of promiscuity associated with molecular scaffolds. For purposes of simplicity, comprehensibility, and practical utility, “promiscuity” is defined in this context simply as multiplicity of positive non-duplicate bioassay results. It is well understood that positive results (hits) may be false positives, where the false indication is due to experimental artifact (e.g. aggregation, reactivity, fluorescence). Yet, such a compound will generally be undesirable regardless of whether its frequent-hitting is due to true or false positives. In this study and in general, different bioassays generally means different targets, where “target” can refer to protein target, or a targeted interaction in a phenotypic screen. This generalization can be rigorously enforced by utilization of assay ontologies such as BAO [30, 31] or BARD [32, 36]. With typical current bioassay data sources, the Badapple definition of statistical promiscuity effectively allows us to identify compounds that are more likely to be non-selective over many assays and many targets. HTS “frequent hitters” may be true or false positives, as confirmed by secondary assays. Regardless, they are likely to be costly “false trails”.

### Why scaffolds?

Scaffolds are used to aggregate data and detect patterns for several reasons: (1) Scaffolds relate analog chemical series, relevant to medicinal chemistry and lead optimization. (2) Data may not exist about a specific compound, but may exist about a closely related compound with common scaffold. (3) “Privileged structures” theory suggests scaffolds often confer bioactivity. We note that the scaffold can contribute to bioactivity directly, via shape or binding interactions, or indirectly, via functionalization potential. In addition, by employing the “HierS” hierarchical scaffold analysis method of Wilkens et al. [33], associations are not limited to the largest scaffold (Bemis–Murko framework [40]).

### Badapple formula for scaffold promiscuity

The Badapple formula is shown below. The promiscuity score (pScore) is a product of three terms, related to substances, assays and samples, each of which needs to be high to produce a high score. Medians are computed across the entire dataset. The use of medians normalizes in such a way that scores are unlikely to be high if the weight of evidence is relatively low.

1 $$\begin{aligned} score & = \frac{{s_{A} }}{{s_{T} + med(s_{T} )}} \times \frac{{a_{A} }}{{a_{T} + med(a_{T} )}} \\ & \quad \times \frac{{w_{A} }}{{w_{T} + med(w_{T} )}} \times 1e5 \\ \end{aligned}$$

where s_T_ = tested substances with scaffold, s_A_ = active substances with scaffold, a_T_ = assays with tested compounds with scaffold, a_A_ = assays with active compounds with scaffold, w_T_ = tested samples with scaffold, w_A_ = active samples with scaffold, med = median.

Experienced practitioners of HTS know that errors occur despite diligent use of best practices. The Badapple formula mitigates the problems of noisy and error-prone data by aggregating across samples and substances. More substances provide more evidence, even when the substances are supposedly identical compounds. More samples tested provide more evidence, even when the samples supposedly contain the same substance, and are subject to the same assay. The score combines and reflects both the “batting average” (BA) and the weight of evidence, so high scores are more likely to indicate a valid pattern. The “BA” concept provides an illustrative term and points to a valid analogy. In baseball analytics (sabermetrics), it is well known that the early BA stats, say from the first month of the season are not predictive of full-season BA [34]. Noisy data requires sufficient sampling to produce evidence. Accordingly, the Badapple score is skeptical of scanty evidence. Table 1 shows the ranges of Badapple scores, as derived from HTS data: low, moderate and high, with corresponding advisories.

**Table 1:** Badapple pScore ranges

pScore range	Advisory
~	Unknown; no data
0–99	Low pScore; no indication
100–299	Moderate pScore; weak indication of promiscuity
300+	High pScore; strong indication of promiscuity

**Fig. 1:**
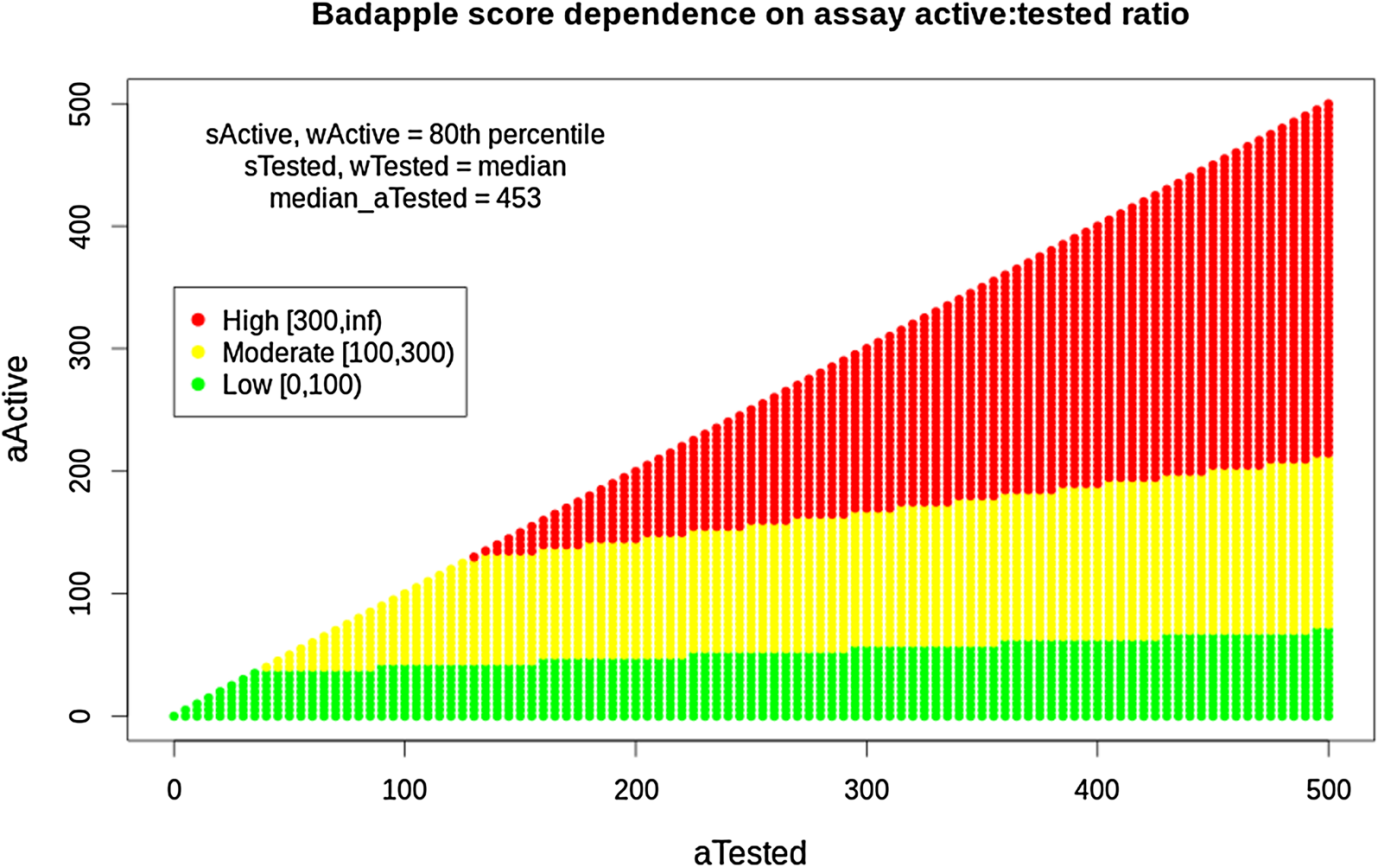
Badapple score dependence on assay-active and assay-tested statistics

In Fig. 1, the color bands indicate the ranges of Badapple scores: low, moderate and high. It is clear that the score is not dependent only on the BA, i.e. ratio of actives to tested, since then the range boundaries would be straight lines through the origin. Rather, when the *aTested* is below a certain threshold (i.e. the weight of evidence is insufficient), moderate or high scores are disallowed. Below another threshold, high scores are disallowed. In this figure, the substance and well terms are held constant. Given the three-way symmetry of the Badapple formula, the corresponding figure for substance and well statistics would reflect the same properties.

### Statistical, Bayesian learning

The Badapple formula is computationally simple, but combines some powerful features. Understanding its relationship to other statistical methods is important for comprehensibility, interpretation and to make best use of the methodology more generally. As one notable comparison, the Badapple formula shares some properties with the Internet Movie Database (IMDb) score used to rank movies in its “Top 250” [35]

2 $$score = \frac{vR}{v + m} \times \frac{mC}{v + m}$$

where R = average rating for the movie, v = votes for the movie, m = minimum votes to be in Top 250 (currently 25,000), C = the mean vote across the whole report (currently 7.0).

In particular, the use of the minimum-votes expression has a similar effect in devaluing high BAs if the weight of evidence is relatively low. IMDb describes their score as a “Bayesian Estimate” (BE). Although neither Badapple nor IMDb makes use of Bayes’ theorem, it may be both justified and explanatory to represent these methods as Bayesian-like. Badapple shares some key features of Bayesian approaches: (1) absence of any assumed probability distribution, and (2) by iterative learning cycles, new data can be used to continually improve the prediction model. Badapple also reflects systematic skeptical bias, meaning restricting the number of high scores using weight of evidence as a marker of confidence, because in the domain of bioassay data analysis, a semi-automated endeavor requiring human judgment, there is a limit to the number of red flags which can be readily processed.

### Knowledge management and “thoroughly conscious ignorance”

While Badapple is robust to noise, it also is completely reliant on both the quality and coverage of the empirical data. If the data does not contain evidence for a given structure, for example, the Badapple score will be zero, representing “no indication”. If the data contains duplicate results, the scores are degraded, since accurate BAs depend on accurate counting. Obviously, multiple positive results in identical assays, or assays for identical targets, are not indicative of promiscuity. The logical implication of being data driven is that without data there is no knowledge. However, to quote James Clerk Maxwell: “Thoroughly conscious ignorance is the prelude to every real advance in science.” By avoiding a prejudiced guess, an algorithm or scientist can be prepared for the arrival of new information.

### BARD and the Badapple plugin

#### Enterprise bioassay analysis

The BioAssay Research Database (BARD) [32, 36] is designed as a resource for bioassay data and particularly its use by researchers in exploratory data analysis and knowledge discovery. Accordingly, BARD includes a powerful and extensible computational platform whereby plugins can be deployed and integrated seamlessly with the BARD public REST API [36]. Badapple was chosen as the first “exemplar” BARD plugin (Additional file 1), based on the scientific value of promiscuity modeling and the direct relevance to bioassay data analysis. The BARD project began in March 2012 and the first version of the Badapple plugin was released in Sept 2012, using training data from PubChem. Subsequent versions of the plugin have used data directly from BARD, and PubChem assay IDs (AID) were replaced by BARD experiment IDs (EID). This migration was a positive step in elevating the semantic rigor of Badapple, toward systematic de-duplication of assays.

### BARD/MLP Badapple data analysis

#### The privileged and notorious few

##### Approach

Rigorous classification of assays and targets in bioassay databases has been a continuing challenge, with efforts ongoing by the projects BAO and BARD. However, MLP assays were selected for scientific merit and therefore tend to be non-redundant. Likewise, MLSMR is a non-redundant screening library by design. Accordingly, for this study, and because Badapple was originally designed for HTS bioassay data analysis, the dataset chosen for analysis consists of MLP HTS assays and MLSMR compounds. Additionally, as the community may be familiar with this data, this well characterized dataset can facilitate interpretation and replication of results.

##### Output scores and statistics

**Fig. 2:**
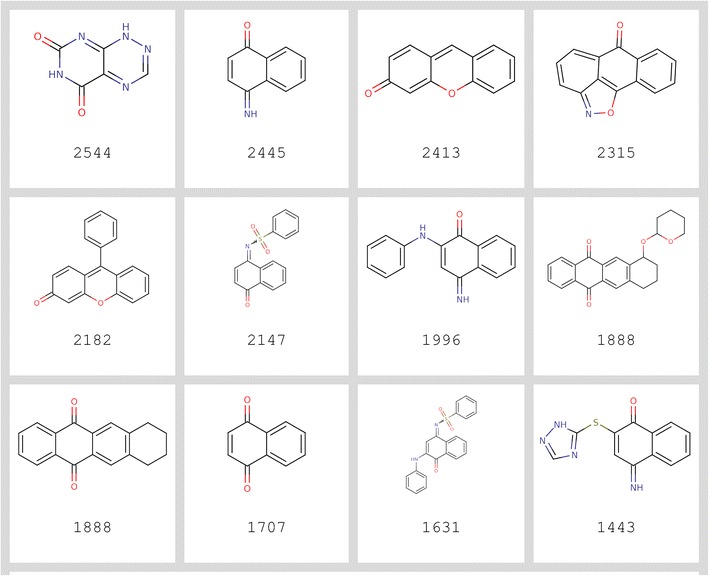
Top promiscuous scaffolds, ranked by Badapple score (see Additional file 2 for full statistics)

Complete Badapple output is provided in the Additional file 2 for all scaffolds in for the BARD-based version “bard1”. These results include computed Badapple scores, plus assay, substance and sample statistics for each scaffold. PostgreSQL databases used in this study are also available via links provided in Additional file 2. The R code used for analysis is also provided, and top scoring scaffolds are presented in Fig. 2.

**Fig. 3:**
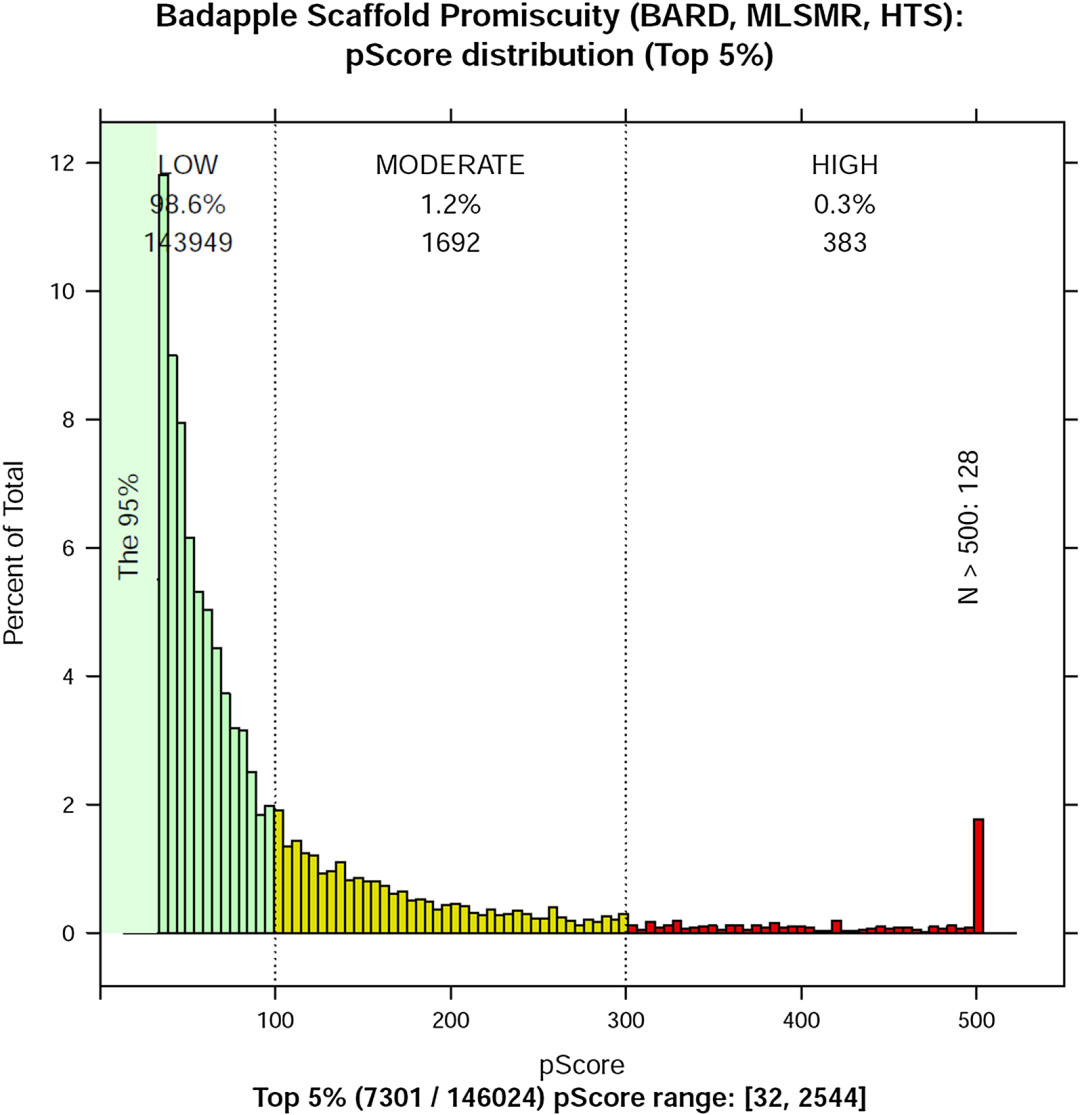
Promiscuity score distribution

The total scaffold count is 146,024, of which 383 (0.3 %) are high scoring, 1692 (1.2 %) are moderate, and the remaining 143,949 (98.6 %) are low scoring. The distribution is shown in Fig. 3. While the cutoff values are arbitrary (and rooted in observed distributions), they are informed by the overall need to efficiently support scientific workflows. Too many “red flags” would increase the chance of false alarms and would themselves be increasingly costly to analyze. Users with sufficient time should not rely on cutoffs but investigate as many high and moderate scores as time permits.

**Fig. 4:**
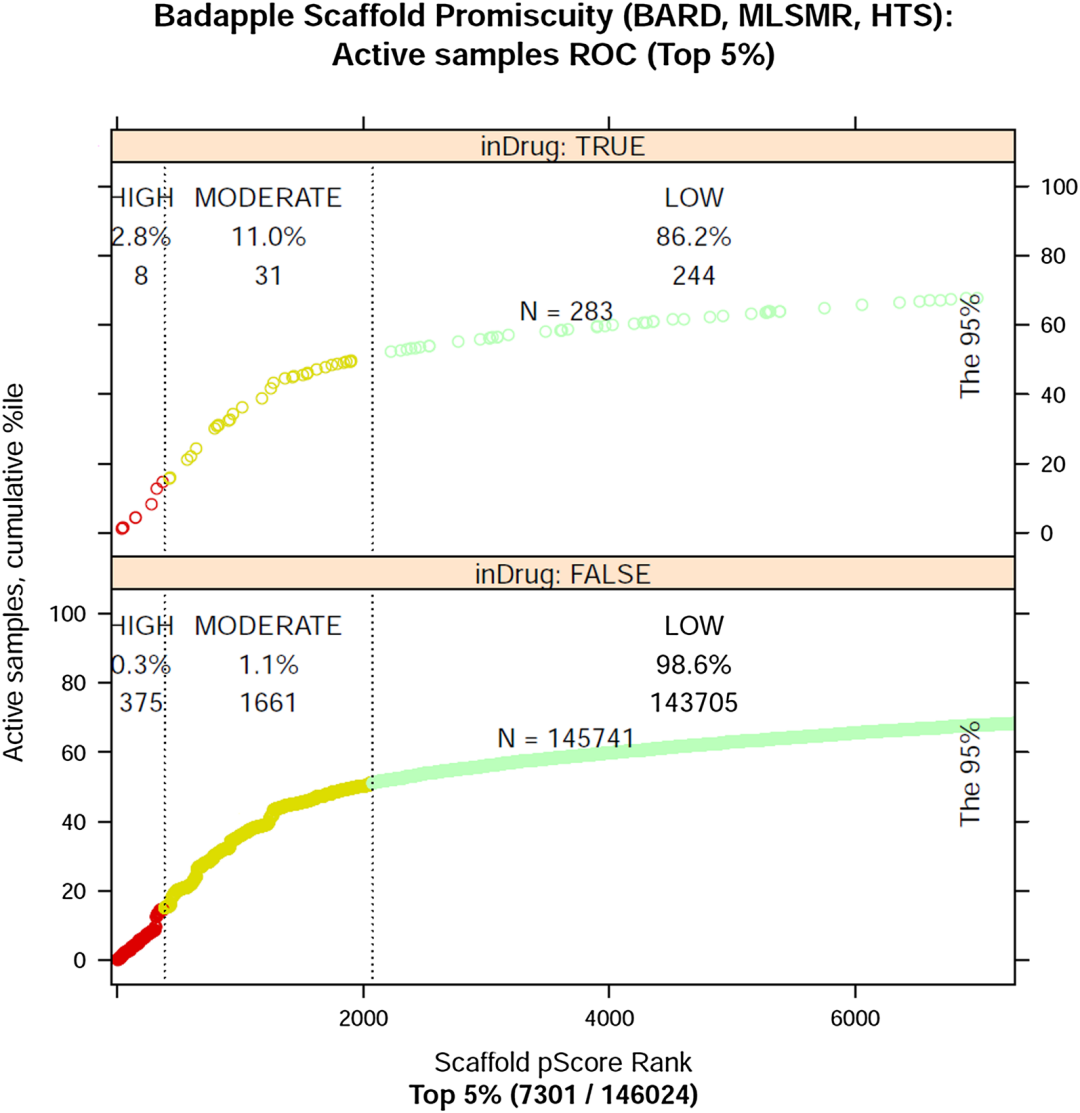
ROC curves, total bioactivity versus ranked top scaffolds for top 5 %

Although there are relatively few high scoring scaffolds, those “privileged” few account for a disproportionate share of the bioactivity in the dataset (i.e. samples deemed active by the assay). Figure 4 illustrates this skewness via receiver operating characteristic (ROC) curves, which plot the percentage of active samples retrieved, with the horizontal axis the top scaffolds (top 5 %) in ranked by score. Overall, 50 % of all bioactivity is associated with 1.4 % (1979) of the scaffolds. (By “50 % of all bioactivity” we mean 50 % of all active samples.) For drug-scaffolds, 50 % of all bioactivity is associated with 2.8 % (54) of the scaffolds. These data support the Badapple approach of identifying a relative few scaffolds for scrutiny, partly because human investigators have limited time, but also because nature appears to confer special properties to a relative few.

### Prediction, validation, and history

#### “Those who fail to learn from history are doomed to repeat it”: George Santayana

The Badapple algorithm is intended as a tool to quickly assess the likely promiscuity of HTS hits or arbitrary compounds of interest. It has been noted that predicting the future is hard. Fortunately, if a future instance is very similar to a prior instance, studying history can suffice. History can also be hard since it requires good information, often a precious commodity. Supervised machine learning (ML) predictive models are also trained on historical data. ML models can be powerful and useful, but are prone to overfitting and often uninterpretable. In contrast, a Badapple score is a statistic, an algebraic function of empirical, historical data.

To validate Badapple we used retrospective datasets to indicate whether scores generated in the past would have been predictive, in other words, consistent with subsequent bioassay findings. The scores and rankings of scaffolds were compared using conventional Pearson correlation and also Spearman rank correlation. Spearman rank is included since for operational purposes where only top-X hits can be investigated, rank may be more important than raw score. We compare scaffolds for which older data yield high scores and new data exists. By comparing the newer, updated scores with the older scores for this subset, we can evaluate the usefulness of Badapple. Refer to Tables 2, 3,4 and 5 for this analysis.

**Table 2:** Badapple datasets

	#scafs	Tested	Nonzero	#assy	Activities	Date	Source
Bard1	146,024	141,642	54,136	510	30M	2013-01	BARD
Bard2	143,098	137,668	52,328	383	46M	2014-06	BARD
Pc1	143,098	141,533	60,200	822	223M	2014-06	PubChem
Pc2	143,098	125,940	50,912	527	113M	2010-12	PubChem

BARD assay counts are experiment counts. Tested means bioassay data exist. Nonzero means tested with nonzero scores

**Table 3:** Badapple dataset comparison: scaffolds in common (total/non-zero)

	Bard2	Pc2	Pc1
Bard1	141,896 41,218	141,629 49,844	141,629 43,951
Bard2		142,817 43,083	142,817 40,463
Pc2			143,087 47,252

**Table 4:** Badapple dataset comparison: PScore correlation, Pearson/Spearman-rank

	Bard2	Pc2	Pc1
Bard1	0.85 0.73	0.95 0.89	0.92 0.85
Bard2		0.86 0.69	0.85 0.72
Pc2			0.96 0.87

**Table 5:** Retrospective comparison of high scores, pc2 versus pc1

Scaffold	pscore_pc2	pscore_pc1	pscore_diff	wTested_diff
	436	395	41	7,234,388
	374	343	31	5,511,793
	443	369	74	2,644,122
	432	392	40	1,701,524
	578	468	110	1,652,980
	618	627	−9	1,019,584
	375	315	60	899,779
	463	366	97	654,420
	365	361	4	524,438
	461	367	94	496,504
	358	303	55	422,280
	805	696	109	376,575
	403	331	72	358,012
	459	487	−28	319,746
	841	721	120	318,307

Scaffolds ranked by number of new activity data (wells tested) after pc1 and in pc2. The small changes in score confirm initial trends, for many new targets and assays

As an additional validation, the global compound set (389,533) was subjected to randomly partitioned fivefold cross validation. For each fold, 1/5th of the dataset served as test set, with 4/5th as training set. By recalculating all scores for the training set scaffolds, using only activity data for training set compounds, we simulated the situation where test set compounds are new and unknown. For each compound, we associate the score from its highest scoring scaffold. For each fold, compounds which previously had associated Badapple scores may not, since the training set is reduced relative to the original Badapple training set. This can be interpreted as a loss of predictive scope. The Pearson correlation was calculated for each fold, for all test compounds, with those unscored assigned a score of zero, to reflect overall predictive power from each training set. Each correlation was approximately 0.9, indicating strong and consistent predictive power. These results are summarized in Table 6.

**Table 6:** K-fold cross validation (K = 5): Ntotal = 389,533, Pearson correlation, all test scores

k	Ntrain	Ntest	Correlation
1	311,540	77,993	0.895
2	311,463	78,070	0.891
3	311,652	77,881	0.898
4	311,565	77,968	0.901
5	311,454	78,079	0.903

### PubChem assays and BARD experiments

Badapple depends on the quantity and quality of input data, where quality includes both accuracy and semantic interpretability. In particular, it should be possible to rigorously resolve whether two assay results reflect the same or different bioactivity phenomena. This challenge has motivated several efforts to improve bioassay annotations, metadata and ontologies, including BAO and BARD. Badapple was designed to accommodate the uncertainties associated with the limited annotations of MLP assays in PubChem. However, it has been well understood that ontology improvements would offer new opportunities for Badapple and related extensions, for example, promiscuity assessment specific to a target class such as G-protein coupled receptors, and automatic extraction of privileged scaffolds from large scale screening data.

### Reconciling big data and small data

Drug discovery campaigns rely on the expert judgment of medicinal chemists for key decisions on which leads to pursue. That expertise derives from formal training, project experience, and community knowledge which may be global or institutional. Cheminformatics and other areas have contributed tools to assist medicinal chemists, and Badapple is such a tool. As such, acceptance and effective use of Badapple will be enhanced if its advice is comprehensible and demonstrably consistent with expert medchem knowledge. To this end the medicinal chemist among us (CAL) evaluated and annotated top scoring scaffolds for chemical issues as drug leads, which are shown in Table 7, accompanied by relevant literature references [37–39].

**Table 7:** Medchem analysis of selected high scoring, promiscuous scaffolds

	Scaffold of well-known toxin toxoflavin present in *Burkholderia glumae*, it was also previously identified by workers at Abbott using the ALARM NMR assay as a thiol trap and cause of false positives in HTS [37]
	Scaffold a 6*H*-anthra[1,9-cd]isoxazol-6-one is known to react with DMSO acting as a nucleophile and undergoes N–O cleavage of the isoxazole ring to form the ring opened anthraquinone, a species known to form covalent adducts [38]
	Scaffold likely made by reaction of orthophenylene diamine with the corresponding furanyl alpha diketone. It could be a false positive if contaminated with the furanyl alpha diketone. It has only weak metal coordinating activity
	Scaffold the synthesis of the tricyclic scaffold by a malononitrile cyclization with a 2-amino-3-formyl-4-oxo-4*H*-pyrido[1,2-a] pyrimidine suggests that the scaffold in this series may be susceptible to Michael attack at what was originally the formyl precursor carbon
	Scaffold is reported to possess strong fluorescence, UV absorbance as well as strong mutagenic activity [39]. The phenyl-2-(2*H*-benzotriazol-2-yl) scaffold is also found in the photostabilizer Tinuvin P

## Conclusions

Badapple is an easy-to-use, readily interpretable algorithm and tool that can assist scientists in navigating a complex scientific and informational landscape. In particular, Badapple is designed for rapid detection of promiscuity patterns in HTS data, using public bioassay evidence. However, Badapple is designed to be trained with additional data, and to detect novel patterns, based on an entirely different chemical library. Compound promiscuity is generally undesirable but must be understood in light of polypharmacology and systems chemical biology. Badapple scores indicate either patterns of true or artefactual promiscuity, either of which can help guide an experimental research project away from “false trails”.

## Methods

**Fig. 5:**
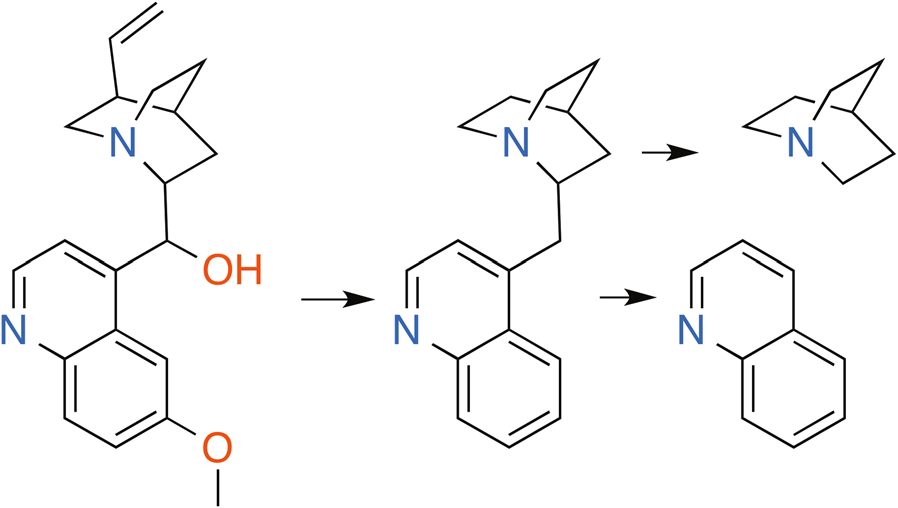
HScaf scaffolds of quinine

The Badapple datasets are all prepared from the MLSMR compound library and HTS bioassays involving at least 20K compounds, to optimize applicability to high-throughput screening methods. The “bard1” dataset was downloaded from BARD in January 2013 and is the default dataset for the analyses herein. BARD was in an early development phase at that time and the dataset is for all intents and purposes equivalent to a PubChem dataset. Accordingly, “bard1” is used in this paper to enhance reproducibility and comprehensibility. The “bard2” dataset was downloaded from BARD in June 2014, and includes fewer assays, reflecting the ongoing annotation and curation efforts at the time. For each dataset the compound library was filtered to remove any salt part and normalize charges. In total, the training data for Badapple was comprised of 389,533 compounds, 438,583 substances, 143,098 scaffolds, 822 HTS PubChem assays, and more than 220 million activity data. In these counts and workflows the terms “compound” and “substance” are defined as by PubChem: compounds as unique chemical structures, identified by CID, and substances as sourced and registered experimental materials, identified by SID. The HierS “hierarchical scaffold” algorithm was used to generate the scaffold library for all compounds. An illustration of the algorithm is shown in Fig. 5. The HierS algorithm perceives for any molecule a hierarchical set of scaffolds, the largest one being equivalent to the “Bemis–Murcko framework” [40], which consists of all ring-systems and linkers. Additional scaffolds represent all the combinations of ring-systems and linkers contained therein. Comprehensive output files and R code used for analysis are provided in supplemental materials (Additional file 2).

**Fig. 6:**
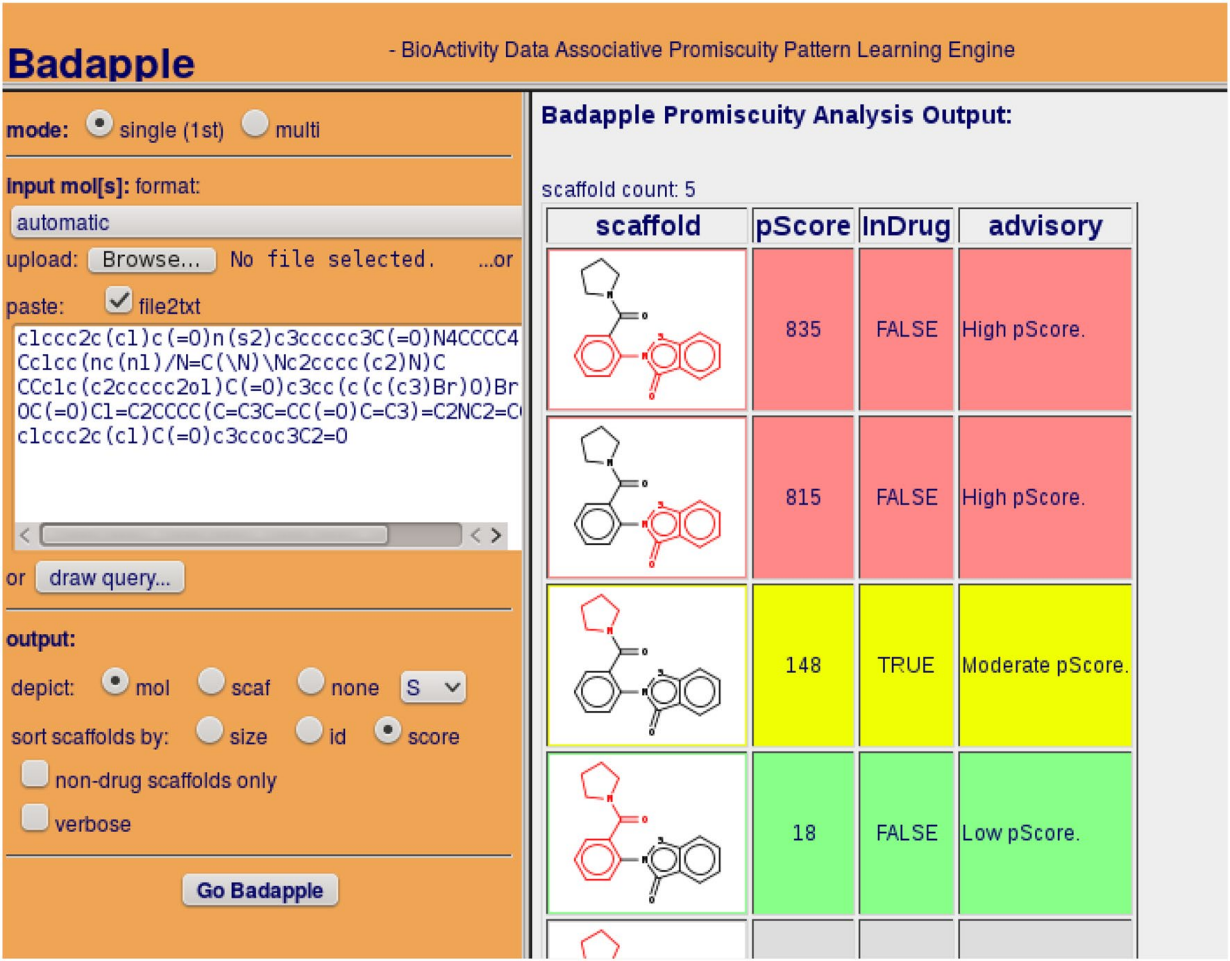
Badapple public web app, available at http://datascience.unm.edu/public-biocomputing-apps

HierS and Badapple were implemented using the ChemAxon JChem Java toolkit [41] with both command line and web interfaces. The HierS implementation has been open-sourced [42]. Badapple is available as a public web app [43] (see Fig. 6).  See also supplementary figure of the Badapple plugin via BARD web client (Additional file 2).

**Fig. 7:**
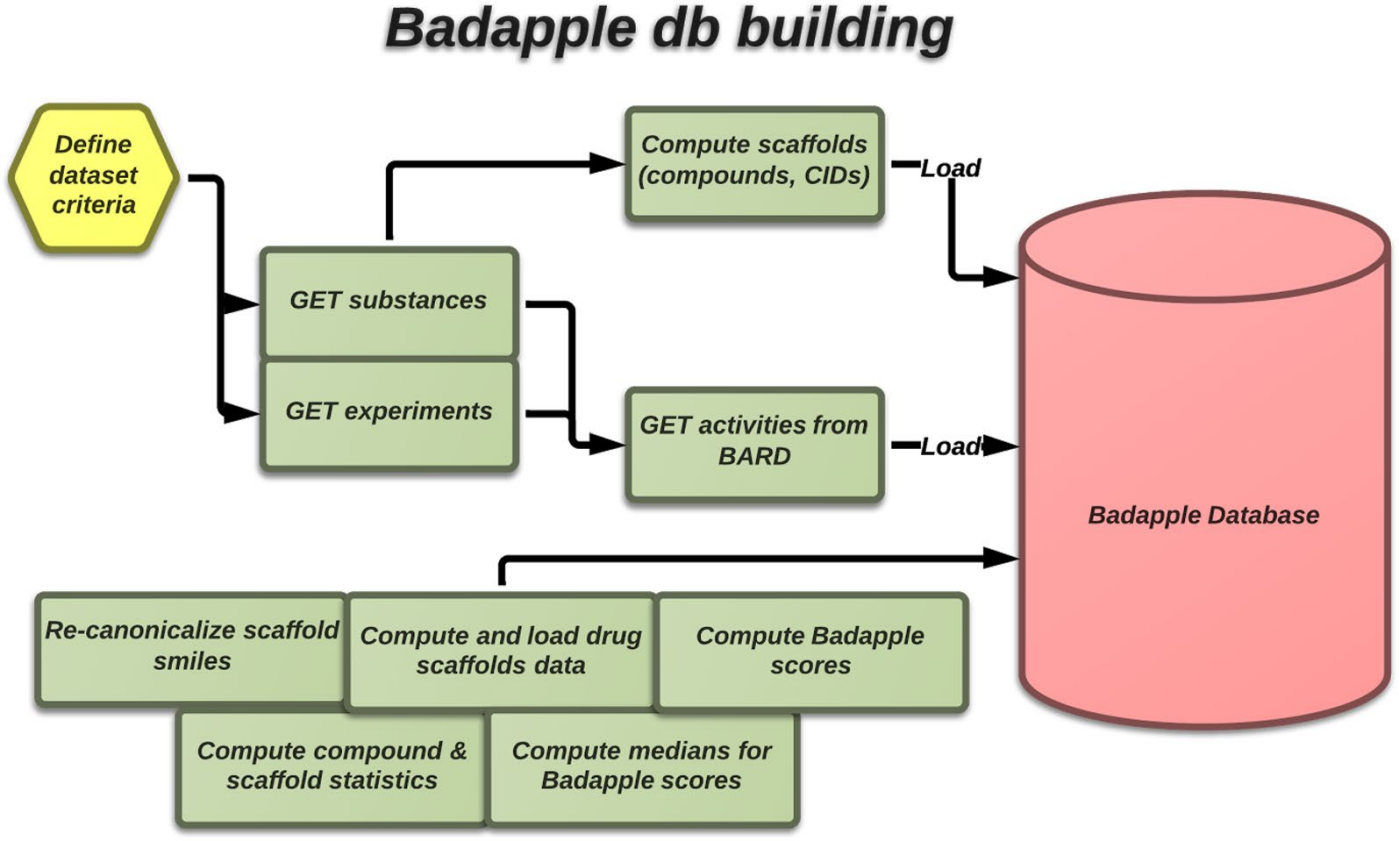
Database workflow

A Badapple database was implemented with PostgreSQL, since rapid scoring, possibly for large numbers of compounds and scaffolds, requires pre-computing and storing aggregate statistics efficiently. OpenChord [44] from gNova was used as a chemical cartridge. Scaffold analysis is performed for all compounds, resulting in links from scaffolds to compounds and substances. Activity data associates substances with an outcome for each sample (typically a well in a multiwell plate) in all the assays in the dataset. Typically there are hundreds of millions of samples. The workflow is outlined in Fig. 7. R was used for statistical analyses.

## Additional files

10.1186/s13321-016-0137-3 Supplementary figures.
10.1186/s13321-016-0137-3 Supplementary files.

## Abbreviations

BA: batting average
BAO: BioAssay Ontology
BARD: BioAssay Research Database
HTS: high-throughput screening
ML: machine learning
MLP: Molecular Libraries Program
MLSMR: Molecular Libraries Small Molecule Repository
Ro5: Rule of 5
SAR: structure–activity relationships
UNMCMD: University of New Mexico Center for Molecular Discovery

## References

[1] Lipinski CA, Lombardo F, Dominy BW, Feeney PJ (1997). Experimental and computational approaches to estimate solubility and permeability in drug discovery and development settings. Adv Drug Deliv Rev.

[2] Rishton GM (1997). Reactive compounds and in vitro false positives in HTS. Drug Discov Today.

[3] Roche O (2002). Development of a virtual screening method for identification of “frequent hitters” in compound libraries. J Med Chem.

[4] McGovern SL, Helfand BT, Feng B, Shoichet BK (2003). A specific mechanism of nonspecific inhibition. J Med Chem.

[5] Seidler J, McGovern SL, Doman TN, Shoichet BK (2003). Identification and prediction of promiscuous aggregating inhibitors among known drugs. J Med Chem.

[6] Teague SJ, Davis AM, Leeson PD, Oprea TI (1999). The design of leadlike combinatorial libraries. Angew Chem Int Ed.

[7] Oprea TI, Davis AM, Teague SJ, Leeson PD (2001). Is there a difference between leads and drugs? A historical perspective. J Chem Inf Comput Sci.

[8] Ajay A, Walters WP, Murcko MA (1998). Can we learn to distinguish between “drug-like” and “nondrug-like” molecules?. J Med Chem.

[9] Sadowski J, Kubinyi H (1998). A scoring scheme for discriminating between drugs and nondrugs. J Med Chem.

[10] Hann MM, Leach AR, Harper G (2001). Molecular complexity and its impact on the probability of finding leads for drug discovery. J Chem Inf Comput Sci.

[11] Hopkins AL, Groom CR, Alex A (2004). Ligand efficiency: a useful metric for lead selection. Drug Discov Today.

[12] Jahnke W, Erlanson D (2006). Fragment-based approaches in drug discovery.

[13] Hajduk PJ, Greer J (2007). A decade of fragment-based drug design: strategic advances and lessons learned. Nat Rev Drug Discov.

[14] Olah MM, Bologa CG, Oprea TI (2004). Strategies for compound selection. Curr Drug Discov Technol.

[15] Hann M, Hudson B, Lewell X, Lifely R, Miller L, Ramsden N (1999). Strategic pooling of compounds for high-throughput screening. J Chem Inf Comput Sci.

[16] Huth JR, Mendoza R, Olejniczak ET, Johnson RW, Cothron DA, Liu Y, Lerner CG, Chen J, Hajduk PJ (2005). ALARM NMR: A rapid and robust experimental method to detect reactive false positives in biochemical screens. J Am Chem Soc.

[17] Yang Y, Chen H, Nilsson I, Muresan S, Engkvist O (2010). Investigation of the relationship between topology and selectivity for druglike molecules. J Med Chem.

[18] Hu Y, Bajorath J (2010). Polypharmacology directed compound data mining: identification of promiscuous chemotypes with different activity profiles and comparison to approved drugs. J Chem Inf Model.

[19] Baell JB, Holloway GA (2010). New substructure filters for removal of pan assay interference compounds (PAINS) from screening libraries and for their exclusion in bioassays. J Med Chem.

[20] Austin CP, Brady LS, Insel TR, Collins FS (2004). NIH molecular libraries initiative. Science.

[21] Kaiser J (2008). Industrial-style screening meets academic biology. Science.

[22] NIH Molecular Libraries Program (2004-2013). https://commonfund.nih.gov/molecularlibraries. Accessed 15 Dec 2015

[23] PubChem Compound. https://www.ncbi.nlm.nih.gov/pccompound?term=pccompound_pcassay_probe[filter]. Accessed 17 Nov 2015

[24] Oprea TI, Bologa CG, Boyer S, Curpan RF, Glen RC, Hopkins AL, Lipinski CA, Marshall GR, Martin YC, Ostopovici-Halip L, Rishton G, Ursu O, Vaz RJ, Waller C, Waldmann H, Sklar LA (2009). A crowdsourcing evaluation of the NIH chemical probes. Nat Chem Biol.

[25] Arrowsmith CH (2015). The promise and peril of chemical probes. Nat Chem Biol.

[26] NIH Molecular Libraries Small Molecule Repository (2004–2013). http://mlsmr.evotec.com/MLSMR_HomePage/. Accessed 15 Dec 2015

[27] MLSMR Excluded Functionality Filters (MLSMR Project Compound Identification) (2007). http://mlsmr.evotec.com/MLSMR_HomePage/identify.html. Accessed 15 Dec 2015

[28] Edwards B, Gouveia K, Oprea T, Sklar L (2014). The University of New Mexico Center for Molecular Discovery. Comb Chem High Thr Screen.

[29] Tseng YJ, Martin E, Bologa CG, Shelat AA (2013). Cheminformatics aspects of high throughput screening: from robots to models: symposium summary. J Comput Aided Mol Des.

[30] BioAssay Ontology (BAO) (2012–2016) http://bioassayontology.org/. Accessed 15 Dec 2015

[31] Vempati UD, Przydzial MJ, Chung C, Abeyruwan S, Mir A, Sakurai K, Visser U, Lemmon VP, Schürer SC (2012). Formalization, annotation and analysis of diverse drug and probe screening assay datasets using the BioAssay Ontology (BAO). PLoS One.

[32] BioAssay Research Database (BARD) (2012). http://bard.nih.gov. Accessed 8 Jun 2015

[33] Wilkens SJ, Janes J, Su AI (2005). HierS: hierarchical scaffold clustering using topological chemical graphs. J Med Chem.

[34] Silver N (2012) The signal and the noise: why so many predictions fail—but some don’t. Penguin Press, New York, p 321

[35] IMDb Votes/Ratings Top Frequently Asked Questions. http://www.imdb.com/help/show_leaf?votestopfaq. Accessed 1 July 2014

[36] de Souza A, Bittker JA, Lahr DA, Brudz S, Chatwin S, Oprea TI, Waller A, Yang JJ, Southall N, Guha R, Schurer SC, Vempati UD, Southern MR, Dawson ES, Clemons PA, Chung TDY (2014). An overview of the challenges in designing, integrating, and delivering BARD: a public chemical-biology resource and query portal for multiple organizations, locations, and disciplines. J Biomol Screen.

[37] Huth JR, Song D, Mendoza RR, Black-Schaefer CL, Mack JC, Dorwin SA, Ladror US, Severin JM, Walter KA, Bartley DM, Hajduk PJ (2007). Toxicological evaluation of thiol-reactive compounds identified using a la assay to detect reactive molecules by nuclear magnetic resonance. Chem Res Toxicol.

[38] Sutter P, Weis CD (1982). Ring opening reactions of 6*H*-anthra[1,9-cd]isoxazol-6-ones and related compounds. J Heterocycl Chem.

[39] Yoshimitsu O, Tetsushi W, Yoshiyasu T, Haruo N, Keiji W (2008). Genotoxic activation of 2-phenylbenzotriazole-type compounds by human cytochrome P4501A1 and N-acetyltransferase expressed in *Salmonella typhimurium umu* strains. Mutat Res.

[40] Bemis GW, Murcko MA (1996). The properties of known drugs. 1. Molecular frameworks. J Med Chem.

[41] JChem 5.8.3, ChemAxon (2012). http://www.chemaxon.com. Accessed 15 Dec 2015

[42] HScaf repository (2012). http://github.com/jeremyjyang/unm-biocomp-hscaf. Accessed 15 Dec 2015

[43] Badapple public web app (2013). http://pasilla.health.unm.edu/tomcat/badapple. Accessed 15 Dec 2015

[44] OpenChord (2009). http://www.gnova.com. Accessed 15 Dec 2015

