# Supplementary material for: Badapple: promiscuity patterns from noisy evidence
Source: J Cheminform. 2016 May 28;8:29. doi: 10.1186/s13321-016-0137-3 (PMC4884375; doi:10.1186/s13321-016-0137-3)
Supplement: Supplementary file 1 — 10.1186/s13321-016-0137-3 Supplementary figures. [file 13321_2016_137_MOESM1_ESM.docx]

| 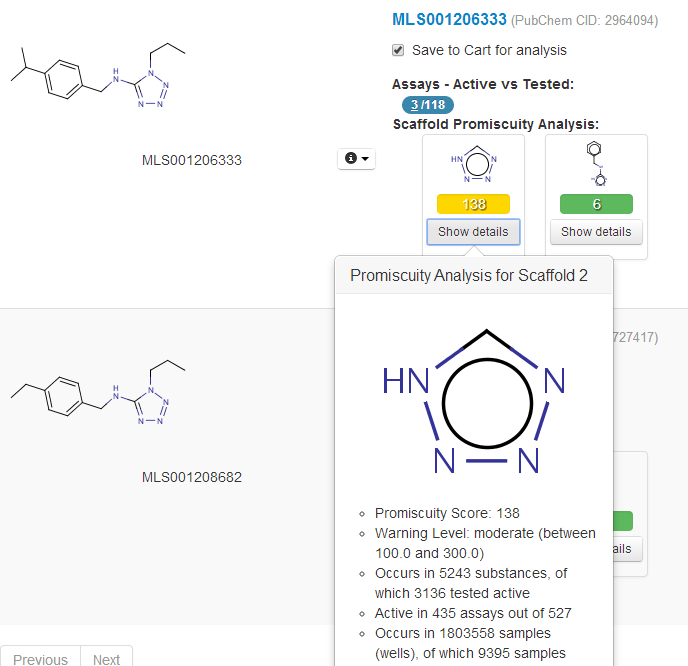 |
| --- |
| **Supplementary Figure 1.** Detail of Badapple plugin via BARD web client. |

| **Scaffold-Assay Activity Ratio** |
| --- |
| **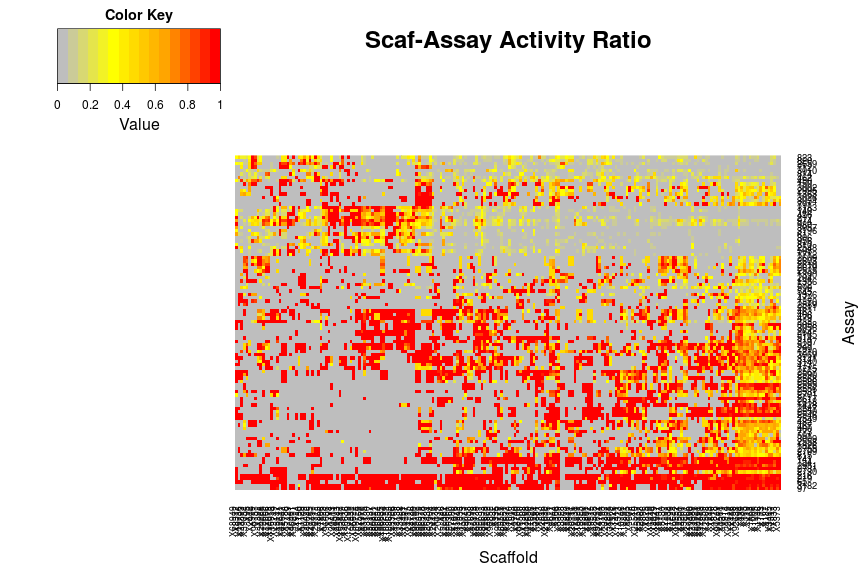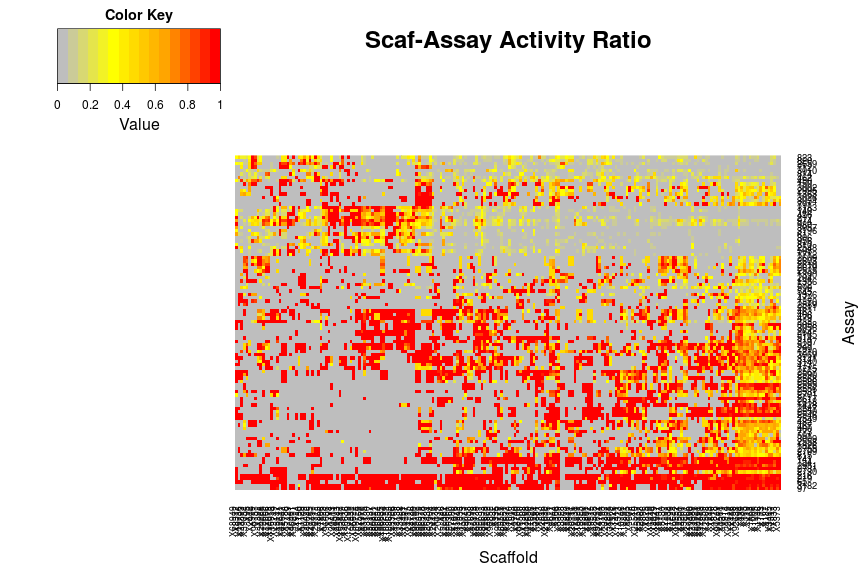** |
| **Supplementary Figure 2.** Scaffold activity by assay, measured by active to tested sample ratio for each assay. Top 200 scaffolds and 100 assays shown. |
